# Considering the Subjective Well-Being of Israeli Jews during the COVID-19 Pandemic: Messaging Insights from Religiosity and Spirituality as Coping Mechanisms

**DOI:** 10.3390/ijerph191912010

**Published:** 2022-09-22

**Authors:** Sidharth Muralidharan, Osnat Roth-Cohen, Carrie LaFerle

**Affiliations:** 1 Temerlin Advertising Institute, Southern Methodist University, Dallas, TX 75205, USA; 2 The Moskowitz School of Communication, Ariel University, Ariel 40700, Israel

**Keywords:** religiosity, spirituality, subjective well-being, terror management theory, health messaging, COVID-19, Jewish, Israel, brief report

## Abstract

Consistent with Terror Management Theory (TMT), COVID-19 has made us question our mortality and past studies have indicated the importance of religiosity to enhance subjective well-being (SWB), however, studies on spirituality’s impact are incomplete. The pandemic has created an environment where both religiosity and spirituality may play a vital role. Israel was selected due to the emergence of Jewish spirituality, a phenomenon that is growing in importance but understudied. In response to these caveats, the current study examines the mediating role played by spirituality on the SWB of the religious during the pandemic. Participants from Israel (*n* = 138) were recruited via Qualtrics’ online panels. Findings showed Jews’ religiosity was important to enhance their SWB, i.e., religious beliefs bring certainty and happiness to one’s life, especially, during the COVID-19 pandemic. More importantly, spirituality mediated the effect of religiosity on SWB, specifically, spirituality was important to enhance the well-being of low religious Jews. Implications for health messaging during a global pandemic are discussed.

## 1. Introduction

The uncertainty surrounding COVID-19 has negatively impacted the subjective well-being (SWB) of many people. SWB is defined as the affective (positive and negative feelings) and cognitive (life satisfaction) evaluation of one’s life [1]. A person who is high on SWB will experience more positive feelings and evaluate their life more favorably. The pandemic has inevitably generated a fear of death, which can truly influence our attitudes and behavior [2]. According to Terror Management Theory (TMT), realization of our mortality can activate self-preservation mechanisms and feelings of terror can be managed through cultural worldviews, self-esteem, and close personal relationships [3]. Cultural worldviews, which ties to this study, are “shared beliefs about reality that provide answers to basic questions about life” [2] and provide organized structure to human perceptions [4]. Religious and spiritual beliefs can mold our cultural worldviews and they have the ability to mitigate mortality salience.

During such trying times, the religious tend to rely on their faith and find solace in religious involvement like prayer. Religiosity is the importance of religion in one’s life [5] and past studies have confirmed a positive relationship with SWB in both western and non-western countries [6,7]. Religious attendance, rituals, and prayers, provide the necessary cushioning against life’s unexpected twists and turns [8,9]. Being involved with one’s religion and having a strong connection with a higher power result in positive evaluation of one’s life and diminished cognitive dissonance [10].

At the same time, apart from religion, spirituality can also play an influencing role but research exploring the ‘spirituality–SWB’ relationship is relatively scant. Spirituality is characterized by one’s relationship with the transcendent and is the pursuit of self-awareness that is realized through interactions with oneself, community, and nature [11]. If religion is a formal and institutionalized form of expression towards the divine then spirituality is a personal pursuit towards understanding life’s meaning and purpose without consideration of the divine [12]. Studies have indicated that the process of spiritual exploration can have a positive influence on one’s well-being [13,14]. In the current study, spirituality has been further conceptualized with a non-divine focus (absence of God) so that it’s distinctiveness from religiosity is enhanced.

While religion and spirituality are empirically and theoretically distinct [15], both contribute to multiple positive human conditions [5]. Previous research demonstrates the influence of religiosity and spirituality as providing support in the face of adversity and toward social rehabilitation, while also being important in the process of self-realization and the development of human abilities [16]. Specific to the current study, the article further highlights how research has found religiosity and spirituality to result in less depression and greater self-esteem [17], better coping skills [18], better physical health [19], and more happiness [20].

In Israel, religion often plays a central role and its social, political, and cultural influences are felt even by the low religious [21], and this unique dichotomy of Israel is the impetus for exploring both religiosity and spirituality. In terms of the country’s religious structure, four major groups exist with varying levels of religiosity. Jews who fall under the ‘traditional’ and ‘secular’ groups are considered as low religious, the ‘religious’ group consists of Jews who are moderately religious, and ‘ultra-orthodox’ are the highly religious [22]. Each group’s Judaist viewpoints are vastly different and the degree of endorsing religious beliefs and the frequency of practicing religious Jewish customs and traditions vary by group [23]. Within this religious landscape, the importance of Jewish spirituality has come to the fore and many reasons can be attributed to its mass acceptance.

Traditional and secular Jews, who occupy the lower end of the religiosity spectrum, consist of more than half of the Jewish population in Israel [24]. The beliefs of a growing segment of spiritual believers in Israel is characterized by a mix of religious Jewish beliefs and spiritual practices [25] and this phenomenon has been termed as ‘Jew Age’ [26]. In order to understand these groups better, a recent study specified two groups of God unbelievers in Israel—‘analytical atheists’ and ‘spiritual-but-not-religious’ (SBNR) [27]. Though both groups had negative attitudes toward religion, the authors’ assumption is that SBNR have the characteristics of those who are ‘Jew Age’ adherents. In other words, SBNR have reported less certainty when it came to their supernatural beliefs and were less outspoken than the religious [27]. Another interesting finding from the study was that these two groups view Judaism as a “cultural worldview and lifestyle,” for example, the Shabbat is celebrated but they do not conform to the religious beliefs behind these practices (p. 55). Furthermore, the pandemic has changed the course of Jewish spirituality’s journey through online platforms and magnified interest in environmental-based spiritual practices [28]. The focus of the current study is not to compare the different Jewish groups but to explore whether religiosity and spirituality enabled Israeli Jews to maintain their well-being during the pandemic.

Understanding how religiosity and spirituality impacts SWB fulfills another purpose for the current study, i.e., it can better inform more effective messaging strategies in relation to health and coping mechanisms. For example, policy makers or health entities that shape marketing communication campaigns during a pandemic can use these research insights when creating public service announcements (PSAs). PSAs are government or non-government sponsored messages that are meant to inform and persuade individuals to adopt desirable behaviors that benefit themselves and society [29]. Early research on COVID-19 has shown PSAs to be effective in combating hesitancy and promoting vaccinations [30] and wearing masks [31]. PSAs may help mitigate high levels of uncertainty and anxiety associated with the pandemic. However, prior to crafting any messages, a deeper understanding of a target audience’ belief system is imperative to heighten message efficacy. Religious beliefs have been shown to impact advertising attitudes and behavior [32] and similar effects can be assumed to be observed for beliefs surrounding spirituality.

**Figure 1:**
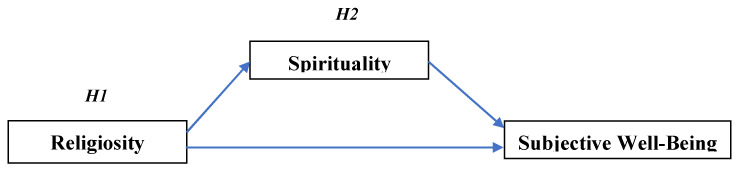
Hypothesized model.

Scarcity in empirical research on the topic and timeliness highlights the importance of the current study. Based on the above discussion on religiosity, spirituality, and SWB, a hypothesized model is presented (see Figure 1) and the following hypotheses are postulated.

**H1.**  *Religiosity will have a positive impact on SWB.*

**H2.**  *Spirituality will play a mediating role and enhance the SWB for those low in religiosity.*

## 2. Materials and Method

Using ‘religion’ as the main quota, Jews (*n* = 138) from Israel were recruited via Qualtrics’ online panels from 12 September to 17 October 2020. Other applied quotas included ‘age’ (25–34: 25%, 35–44: 25%, 45–54: 25%, and 55+: 25%) and ‘sex’ (male and female, at 50% each). The survey was in Hebrew and back translation technique confirmed the validity of the translated questionnaire [33]. Respondents began the survey after agreeing to the informed consent which had first been reviewed and approved by the University’s research ethics committee (ref# H20-125-MURS). They answered questions regarding their religiosity, spirituality, and SWB. Demographic information was collected (Mean age was 34.36, 50.7% were women, and 37.7% had a bachelor’s degree), and respondents were incentivized for their participation.

### Measures

Religiosity (α = 0.90; [34]) and spirituality (α = 0.94; [35]) were measured on a six-point Likert scale (1-strongly disagree to 6-strongly agree). For this study, each item of the spirituality scale began with “Without the presence of God,” in order to be distinct from religiosity. SWB (α = 0.94; [36]) was measured on a six-point semantic differential scale and respondents were asked to describe how they felt about their present life.

## 3. Results

Analyses were conducted using SPSS. To explore the level of religiosity and spirituality, a paired samples *t*-test was conducted. Participants were more spiritual (M = 3.46, SD = 1.46) than religious (M = 3.03, SD = 1.42) (*t* (137) = −2.11, *p* < 0.05). Participants’ subjective well-being during the pandemic was moderate (M = 3.71, SD = 1.15).

To test for multicollinearity, a linear regression was conducted where religiosity and spirituality were entered as independent variables, while SWB was the dependent variable. Tolerance values for both were at 0.872, indicating an absence of multicollinearity between religiosity and spirituality [37].

Using Jamovi, an open-source statistical package, a confirmatory factory analysis (CFA) was conducted with items for religiosity, spirituality, and SWB entered altogether. Results showed that the factor loadings were significant (*p* < 0.001) and the resulting model had adequate good fit (χ^2^ (186) = 356, *p* < 0.001; TLI = 0.92; CFI = 0.93, SRMR = 0.06, RMSEA = 0.08 (90% CI of 0.07 to 0.09)) [38]. The composite reliability (CR) and average variance extracted (AVE) for each factor met the minimum criteria of 0.70 and 0.50, respectively. The final scales were reliable and the items are listed in Table 1.

To test the main hypotheses, Model 4 of the PROCESS macro was used with a bootstrapping procedure of 5000 samples [39]. Religiosity was entered as the independent variable, spirituality was the mediator, and SWB was the dependent variable. Findings showed that religiosity had a negative impact on spirituality (*B* = −0.36, SE = 0.08, *p* < 0.001). Religiosity (*B* = 0.21, SE = 0.07, *p* < 0.01) and spirituality (*B* = 0.22, SE = 0.07, *p* < 0.01) had a positive impact on SWB. Spirituality mediated the effect of religiosity on SWB (*B* = −0.08, SE = 0.03, 95% CI = −0.1576 to −0.0226). Based on the above results, H1 and H2 were supported.

## 4. Discussion

According to Terror Management Theory (TMT), the pandemic has instilled a fear for our mortality and to assuage these feelings, religiosity and spirituality emerged as coping mechanisms. The aims of this study were to address two major gaps in the literature—(a) confirming religiosity as a separate coping mechanism from spirituality, and (b) exploring the impact of spirituality among Israeli Jews during the pandemic. By sampling Jewish participants from Israel, the researchers set out to test religiosity’s impact on SWB, as well as, the mediating effects of spirituality.

As hypothesized, religiosity was found to have a positive impact on one’s well-being. Being involved in one’s religion and following traditional beliefs and practices brought comfort and hope in troubled times. This finding is in accordance with prior studies that have shown God to serve as a secure base for theological exploration and that the connection between religious coping and well-being in times of stress are stronger among the more religious [18,40]. In addition, this link has been found especially powerful among individuals experiencing more stressful conditions that challenge them beyond their immediate resources (e.g., death, terminal illness) [41]. According to TMT, the pandemic and its association with mortality, has activated a self-preservation mechanism [2,3], where individuals are seeking a sense of certainty through their religious beliefs and practices. Indeed, an unpredictable health crisis, such as COVID-19, can intensify the use of religion [42] and connection with a higher power provides the means to achieve a positive life assessment [12]. More importantly, religiosity was found to have a negative impact on spirituality, but spirituality had a positive impact on SWB. In other words, Jews with low religiosity (vs. high) will achieve enhanced life satisfaction through spirituality.

Spirituality has been found to be a separate buffer against mortality salience among the religious and non-religious [43] and the current study highlights its importance on spiritual Jews’ SWB. The avoidance of spirituality among the highly religious can be attributed to the connection between *spirituality* and ‘absence of God,’ which suggests that perspectives on spirituality differs with levels of religiosity. For a highly religious Jew, the existing predetermined perspective of traditional spirituality is that it is closely related to religion [44,45], describing the meaningful exploration of the inner self in relation to the broader reality, includes the presence of God. This is supported by earlier studies arguing Jewish spirituality involves the recognition of the role of “Hashem” (Jewish God) in the way Jews live their lives and involves loyalty to ethical norms [46]. The most important value is “choose life” which nowadays is even more relevant than ever. Jewish spirituality emphasizes the individual or communal longing toward the divine and life of sacredness, or “life in the presence of God” [47]. Jewish spirituality involves infusing the physical world with meaning and seeking the divine in the ordinary [46].

On the contrary, low religious Jews’ schema of values, expectations, and behaviors does not necessarily include a higher power, demonstrating incongruity with their beliefs and a different perspective of spirituality. Spiritual followers or ‘Jew Age’ of Israel tend to combine religious beliefs with spiritual practices [25,26] and the present findings tend to reflect the SBNR group who prefer to follow religious practices or rituals as a lifestyle but do not endorse the associated religious beliefs [27]. Spiritual vibrancy and experimentation, coupled with individualism, emerged in the last decade [48], and using traditional spirituality patterns (i.e., seeking life’s meaning and purpose), in the absence of God, may help to process the relevancy or congruency of the information and fit their existing schema [49], resulting in enhanced SWB. Depending on one’s level of religiosity, these findings reveal a clear distinction in how SWB is realized and is an important finding, considering that nearly half of the Jewish population are secular or the least religious [22]. For instance, 79% of the secular people in Israel say that religion is ‘not too important’ or ‘not at all important’ in their lives [50].

The current findings have important managerial implications for religious and spiritual institutions who are reaching out to their constituents during the COVID-19 pandemic. Religious institutions in Israel can use religiosity to segment their target market and can reach out to their members highlighting the importance of religiosity in their lives. Health messaging that emphasizes how prayer and God can help improve SWB would prove to be effective. Jewish spiritual institutions or education centers (e.g., https://www.jewishspirituality.org accessed on 23 August 2022) that are invested in promoting spirituality can focus on the less religious. Health messages that emphasize the importance of realizing life’s meaning and purpose, finding inner peace, and enabling self-healing can bolster SWB. Furthermore, for spirituality to work with this group, it is important for health messages to avoid any specific references to God and would be prudent to place spiritual goals and benefits as the basis of the message, while downplaying promotional references. While the current study sampled within Israel, the findings could provide insights to consider about the intersection of well-being in relation to effective communication efforts across other religions. However, more research should be undertaken.

### Limitations and Future Research

The study has limitations that need to be discussed. First, due to the exploratory nature of the study, the small sample size was appropriate [51], however, to enhance generalizability future research could procure a larger sample. Second, the current study sampled Jews in general and not from any specific religious groups. Future researchers could sample from the major Jewish religious groups (traditional, secular, religious, and ultra-orthodox) and test the hypothesized model. Between-group comparisons, both among Jews but also across religions, can give researchers a better understanding on how religiosity and spirituality can help enhance SWB for each group. Finally, the life satisfaction scale used to measure SWB tapped into the cognitive dimension [36] and future research could add other scales that measure the affective component [1]. For example, the ‘Scale of Positive and Negative Experience’ (SPANE) helps assess both positive and negative feelings in one’s life [52]. This study’s model (see Figure 1) can be extended to include the affective component of SWB, thereby, adding more depth to the findings.

## 5. Conclusions

Subjective well-being (SWB) has declined with the onset of the COVID-19 pandemic and with fear of contracting the virus, many have turned to religiosity and spirituality to find solace and certainty. Israel was selected due to the emergence of Jewish spirituality, a phenomenon that is growing in importance but understudied. Understanding how religiosity and spirituality impacts SWB fulfills another purpose for the current study, i.e., it can better inform more effective messaging strategies in relation to health and coping mechanisms. The findings have implications for health messaging during a global pandemic. Religious goals embedded in health messages maybe more impactful on highly religious Jews, while health messaging highlighting spiritual goals maybe more impactful on low religious Jews.

## Figures and Tables

**Table 1:** Confirmatory factor analysis.

Factor Items	Loadings	CR	AVE	M (SD)
*Subjective Well-Being*		0.93	0.63	
Lonely-friendly	0.757			3.73 (1.47)
Empty-full	0.774			3.63 (1.41)
Does not give much chance-brings out the best in me	0.801			3.89 (1.37)
Discouraging-hopeful	0.845			3.70 (1.45)
Disappointing-rewarding	0.851			3.54 (1.35)
Miserable-enjoyable	0.804			3.79 (1.29)
Useless-worthwhile	0.817			3.91 (1.36)
Worried-calm	0.781			3.66 (1.53)
Boring-interesting	0.722			3.63 (1.46)
*Spirituality*		0.94	0.70	
Without the presence of God, I can still have a sense of harmony or inner peace.	0.655			3.46 (1.71)
Without the presence of God, I can still have the ability for self-healing.	0.811			3.61 (1.73)
Without the presence of God, my innerness or an inner resource still helps me deal with uncertainty in my life.	0.931			3.77 (1.69)
Without the presence of God, even when I feel discouraged, I can still trust that life is good.	0.910			3.50 (1.69)
Without the presence of God, my life still has meaning and purpose.	0.852			3.83 (1.66)
Without the presence of God, I can still experience moments of peace in a devastating event.	0.824			3.04 (1.73)
Without the presence of God, I can still go to a spiritual dimension within myself for guidance.	0.854			3.01 (1.63)
*Religiosity*		0.90	0.62	
I am very religious.	0.834			2.42 (1.59)
Religious values are more important than material things.	0.720			3.17 (1.68)
If people were more religious, this would be a better world.	0.838			2.62 (1.69)
My religion is very important to me.	0.794			3.69 (1.83)
I pray regularly.	0.835			2.38 (1.66)

## Data Availability

The data that support the findings of this study are available on request from the corresponding author. The data are not publicly available due to restrictions, e.g., containing information that could compromise the privacy of research participants.
